# Supplementary material for: FcγR engagement reprograms neutrophils into antigen cross-presenting cells that elicit acquired anti-tumor immunity
Source: Nat Commun. 2021 Aug 9;12:4791. doi: 10.1038/s41467-021-24591-x (PMC8352912; doi:10.1038/s41467-021-24591-x)
Supplement: Supplementary file 1 — Supplementary Information [file 41467_2021_24591_MOESM1_ESM.pdf]

SUPPLEMENTARY INFORMATION

FcγR engagement reprograms neutrophils into antigen cross-presenting cells that elicit acquired anti-tumor immunity

Vijayashree Mysore[1], Xavier Cullere[1], Joseph Mears[2,3,4], Florencia Rosetti[5], Koshu Okubo[1#], Pei X. Liew[1#], Fan Zhang[2,3,4], Iris Madera-Salcedo[5], Frank Rosenbauer[6], Richard M. Stone[7], Jon C. Aster[1], Ulrich von Andrian[8], Andrew Lichtman[1], Soumya Raychaudhuri[2,3,4,9], Tanya N. Mayadas[1,10]

This PDF file includes:

SUPPLEMENTARY FIGURES

Supplementary Figure 1 (associated with Figure 1)
Supplementary Figure 2 (associated with Figure 1)
Supplementary Figure 3 (associated with Figure 1)
Supplementary Figure 4 (associated with Figure 2)
Supplementary Figure 5 (associated with Figure 5)
Supplementary Figure 6 (associated with Figure 6)
Supplementary Figure 7 (associated with Figure 7)
Supplementary Figure 8 (associated with Figure 8)
Supplementary Figure 9 (associated with Figure 8)
Supplementary Figure 10 (associated with Figure 8)
Supplementary Figure 11 (associated with Figure 8)
Supplementary Figure 12 (associated with Figure 8)
Supplementary Figure 13 (associated with Figure 8)
Supplementary Figure 14 (associated with Figure 9)

SUPPLEMENTARY TABLES

Supplementary Table 1. Demographic and clinical characteristics of SLE study population.
Supplementary Table 2. Clinical characteristics of samples from patients with myeloid neoplasias.
Supplementary Table 3-6. Source tables for antibodies used for flow cyometry.

Supplementary Figure 1

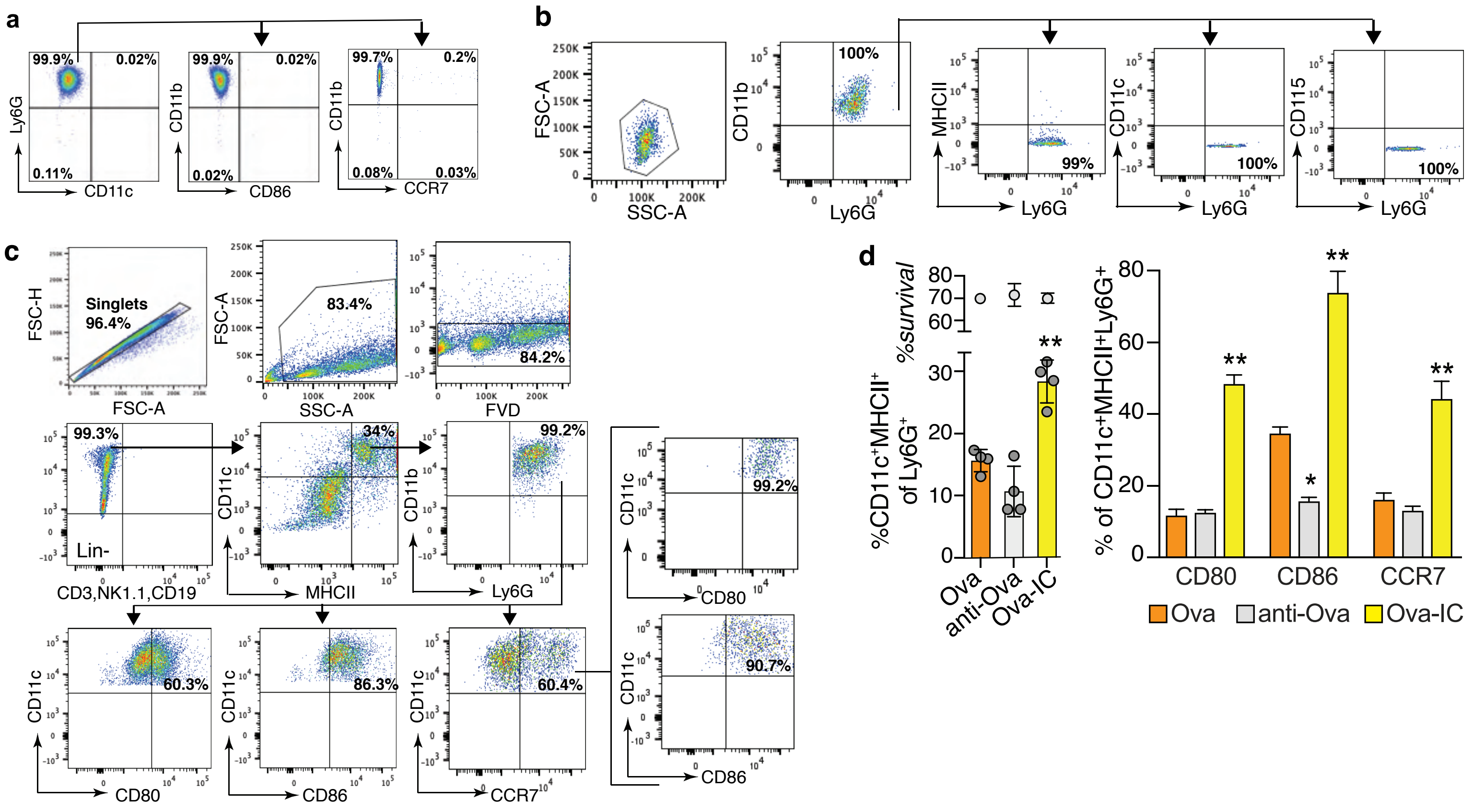

	MHCII	CD80	CD86	CCR7	MHCII/CD80 CD86/CCR7
CD11c+	85.9 ±1.45	93.1± 5.8	69.0± 8.8	78.5 ± 7.9	35.1 ± 0.5
CD11c-	6.1 ±1.0	3.5± 1.1	4.1± 2.1	3.3± 1.1	0.0 ± 0.0

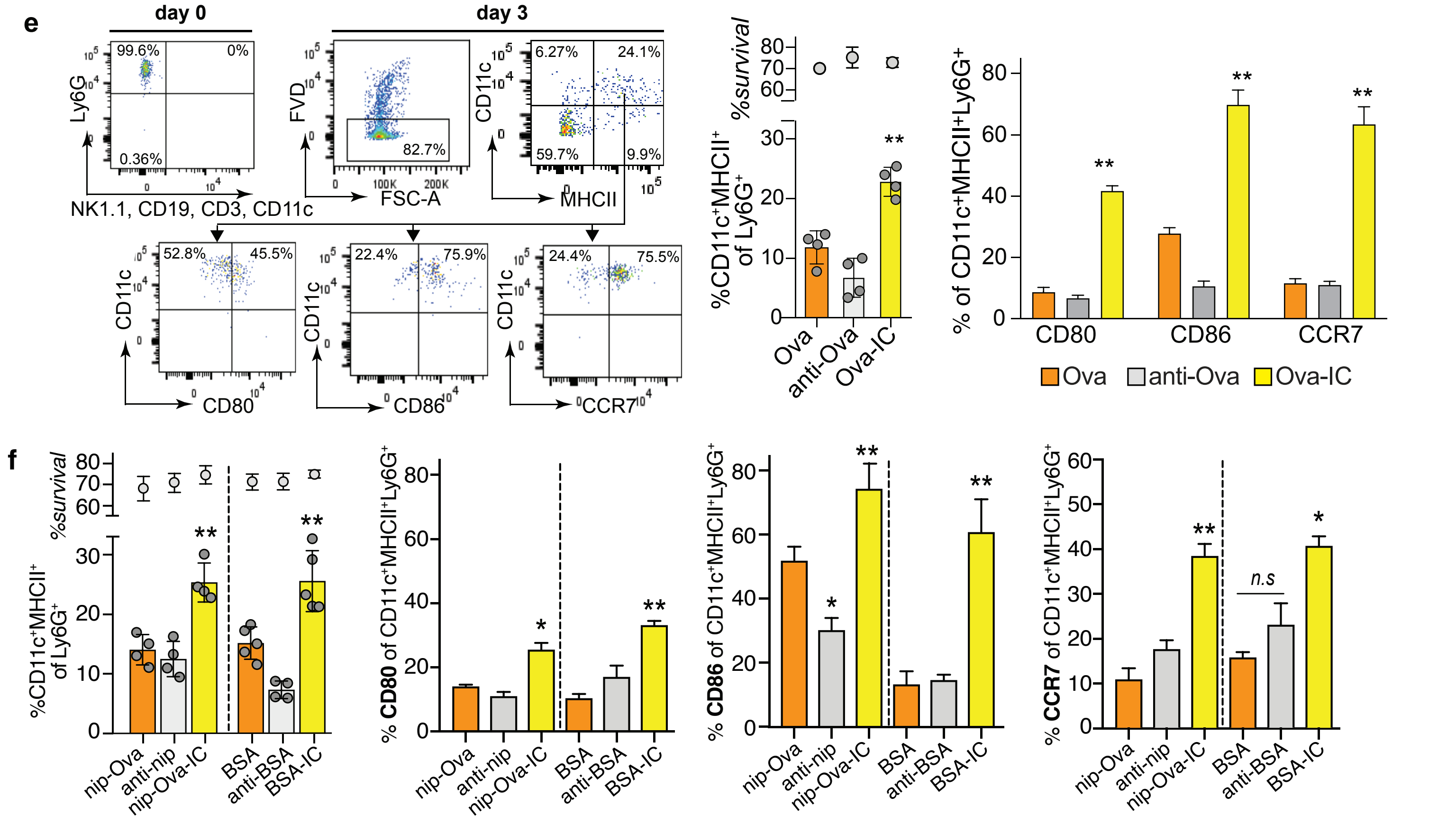

Supplementary Figure 1, cont'd

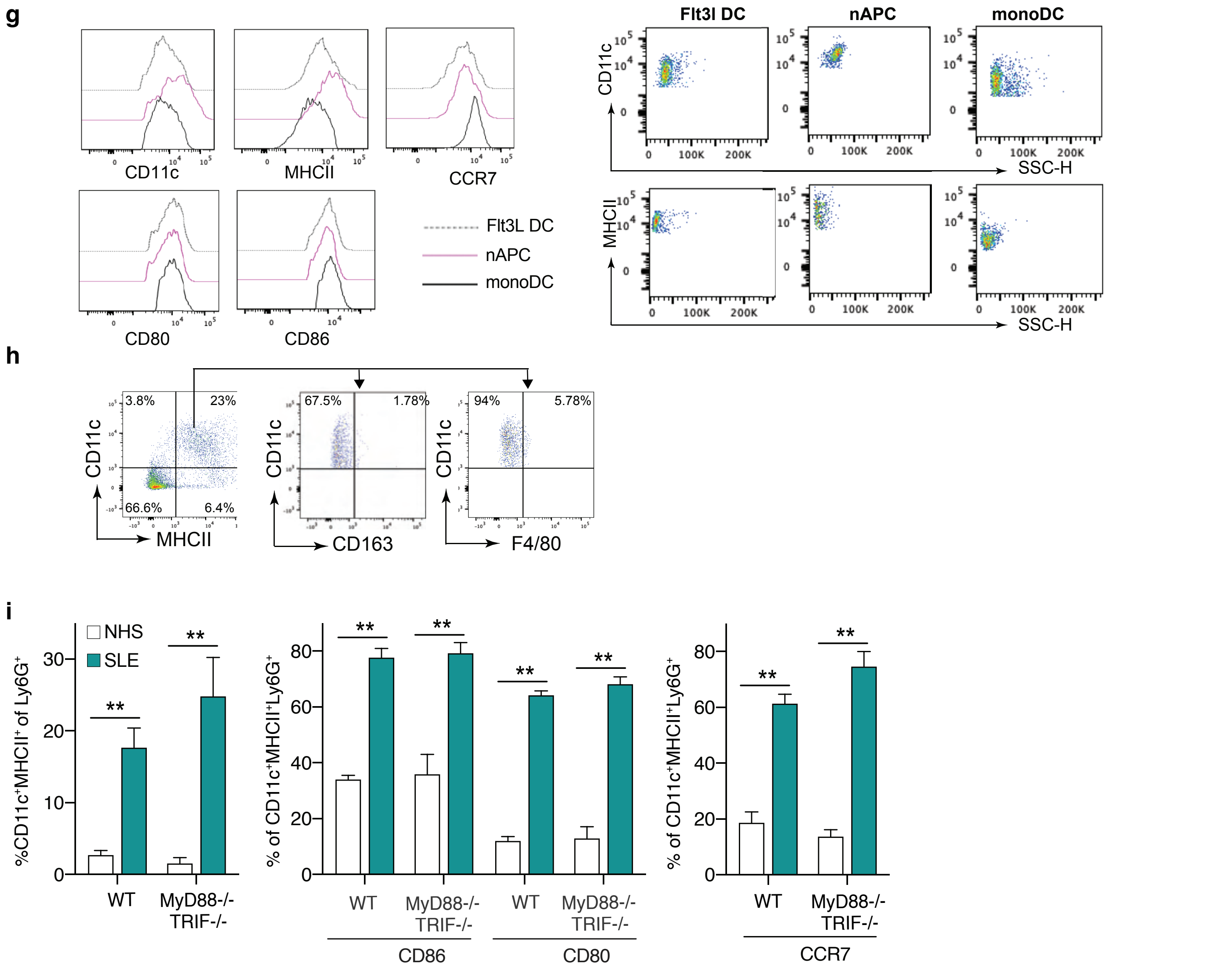

Supplementary Figure 1 (associated with Figure 1). Gating strategies for flow cytometry data analysis and nAPC characterization. a) Flow cytometric plots assessing purity of murine peripheral blood neutrophils, representative of 10 independent experiments with similar results. **b)** Flow plots assessing purity of bone marrow neutrophils from G-CSF treated mice and stained with indicated markers. **c)** Representative FACS gating strategy for nAPCs derived from Ova-IC treated neutrophils. Table shows percent of Ly6G+ population that is positive or negative CD11c expressing the indicated markers. **d)** After 3 days, cells from (c) were evaluated by flow cytometry for survival and acquisition of CD11c and MHCII; the positive population was further analyzed for CD80, CD86 and CCR7. **e)** FACS plots for sam-ples at days 0 and 3 from non-G-CSF treated BMNs (left panel). Bar graphs of percent of Ly6G^{+} that acquire CD11c, MHCII and additionally CD80, CD86 and CCR7 after 3 day in culture (right panel). **f)** nAPCs were generat-ed from BMNs using Nip-Ova/anti-Nip ICs (nip-Ova-ICs), BSA/anti-BSA ICs (BSA-ICs) or their individual compo-nents. After 3 days, Ly6G^{+} cells were analyzed for CD11c^{+}MHCII^{+} and then further for CD80, CD86 and CCR7. **g)** Vertical half offset (left panel) of mean fluorescence intensities (MFI) and plots (right panel) for the indicated mark-ers on Ova-IC-nAPC, splenic monocyte derived DCs (monoDC) and Flt3L-induced splenic DCs. **h)** Ova-IC gener-ated nAPCs expressing CD11c and MHCII were analyzed for the macrophage markers CD163 and F4/80. **i)** BMNs from WT and MyD88$^{-/-}$ TRIF$^{-/-}$ mice were treated with Normal (NH) or SLE (SLE) sera and the percent nAPC gener-ated was determined as in f). Data is mean ± s.e.m. d, e, f) One-way ANOVA and Dunnett's multiple comparison test; i) Student t-test for unpaired comparisons with Dunn-Bonferoni was used to determine significance. *P<0.05 and **P<0.005.

Supplementary Figure 2

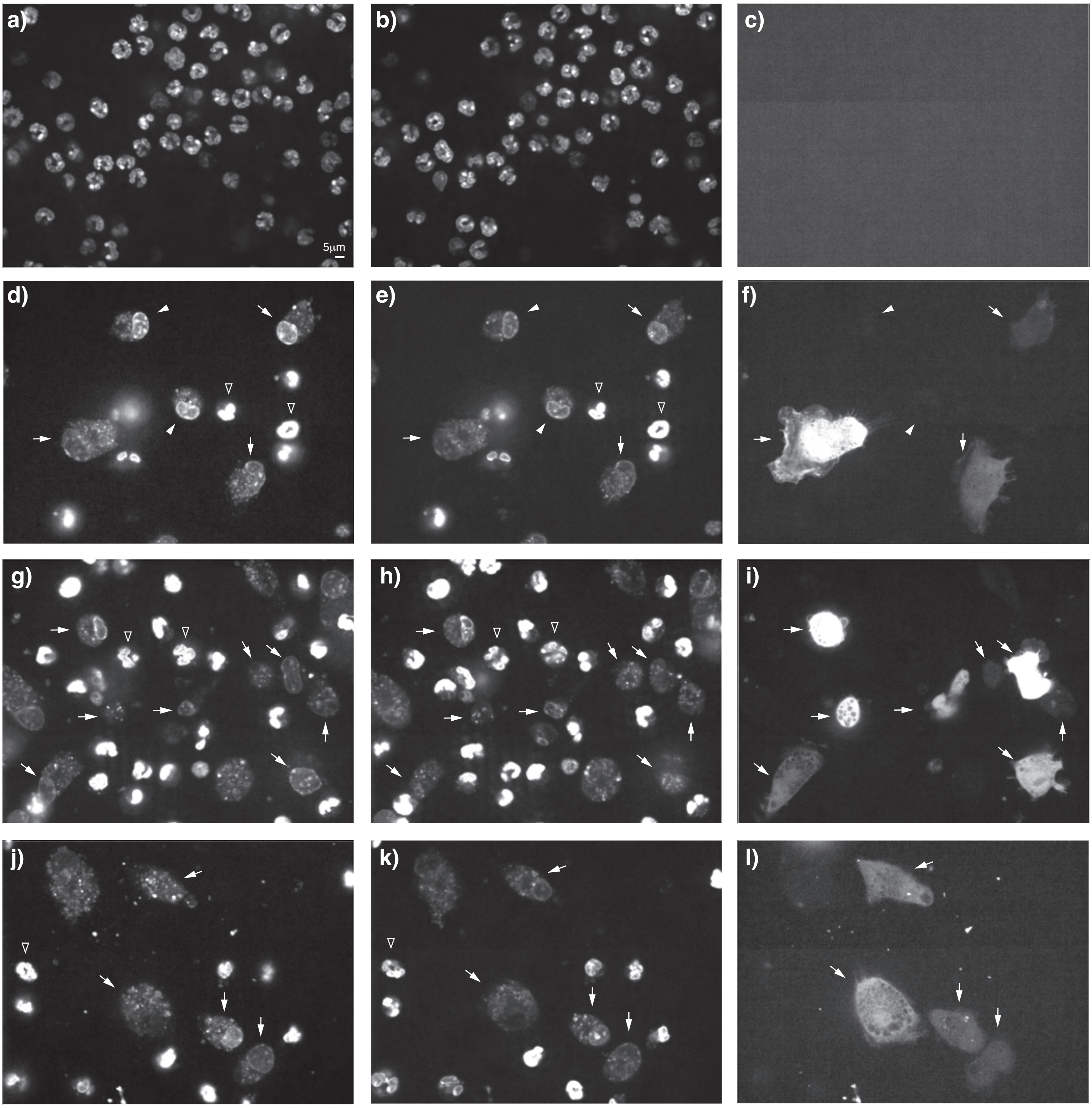

Supplementary Figure 2 (associated with Figure 1). Confocal imaging of SLE-immune complex induced neutrophil to nAPC con-version. Representative montages of CD11c-YFP reporter mouse blood neutrophils, showing YFP (c, f, i, l) and Cy5 fluorescence channels (a-b, d-e, g-h, j-k) at two different z-stacks to delineate nuclear morphology. Open head arrows indicate unconverted neutrophils, closed arrows indicate YFP-positive cells with mononu-clear-like nuclei. Closed arrowheads in d-f indicate additional mononuclear cells with low CD11c-YFP signal. Subpanels (a-f) are replicates of Figure 1 with additional z-stacks for Cy5 at day 0 (a-b) and day 3 (d-e). Sub-panels (g-l) are day 3 images from an independent sample. All images are the same magnification. Scale bar (a) = 5μm.

Supplementary Figure 3

a

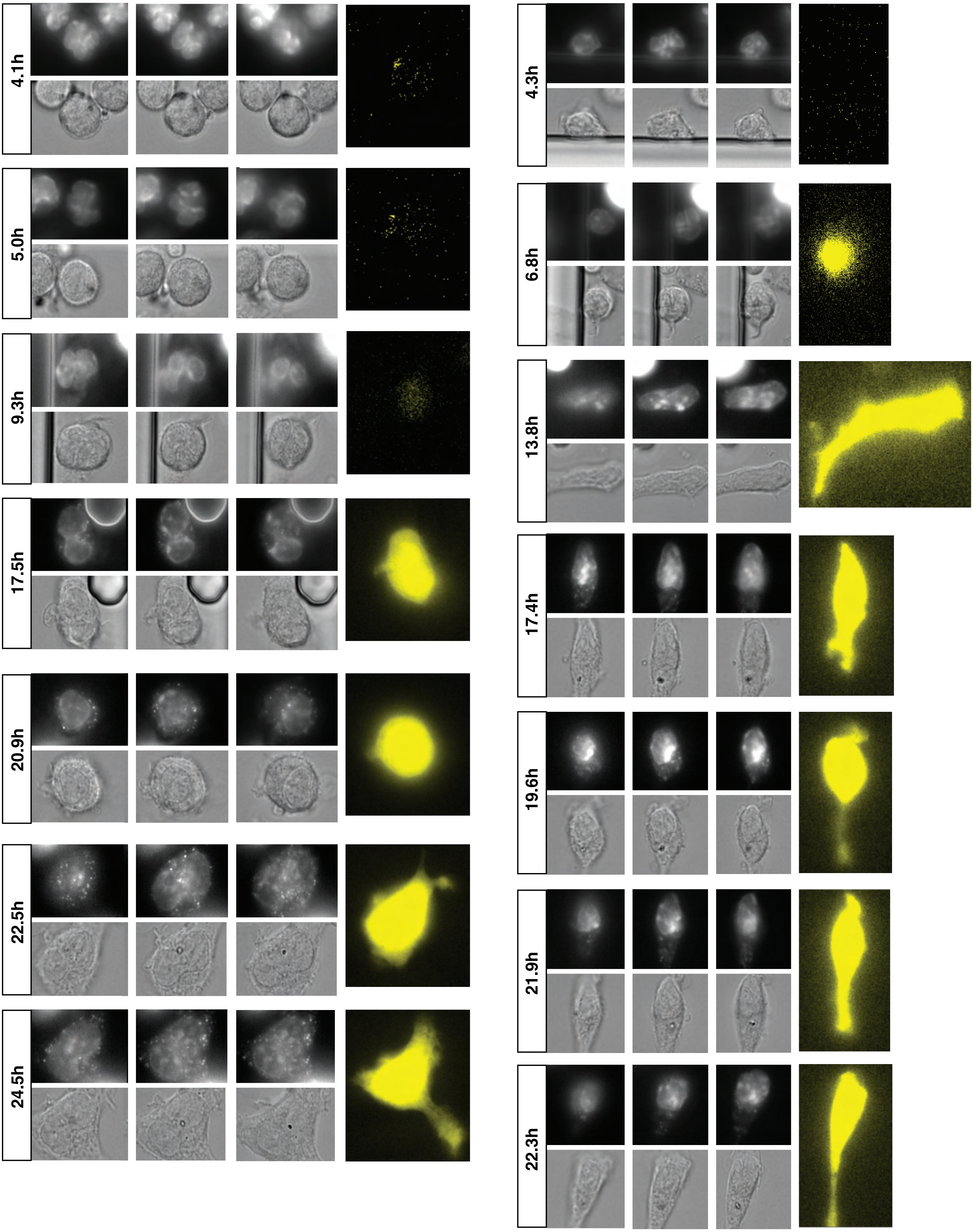

Supplementary Figure 3 cont'd

b

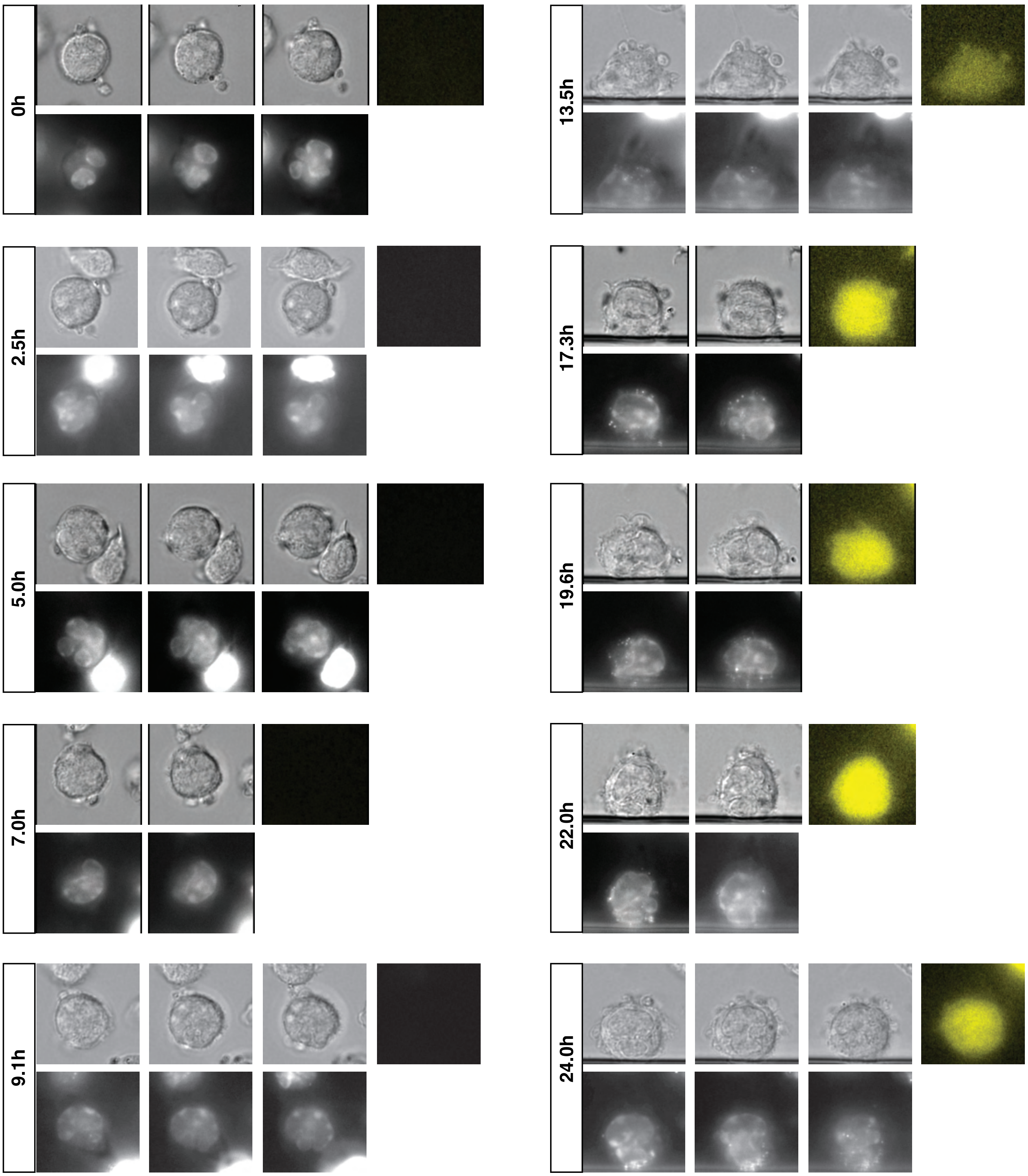

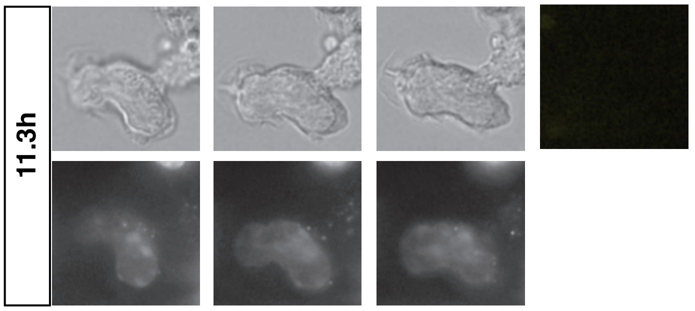

Supplementary Figure 3 (associated with Figure 1). Live cell imaging of SLE-im-mune complex induced neutrophil to nAPC conversion. a-b) Montage of bright field and Cy5 illustrating nuclear and cell morphology and YFP channel representative of CD11c induction at different focal planes and time points after SLE-IC stimulation during live cell imaging. Images tracking two individual cells **(a)** and one cell **(b)** are shown.

Supplementary Figure 4

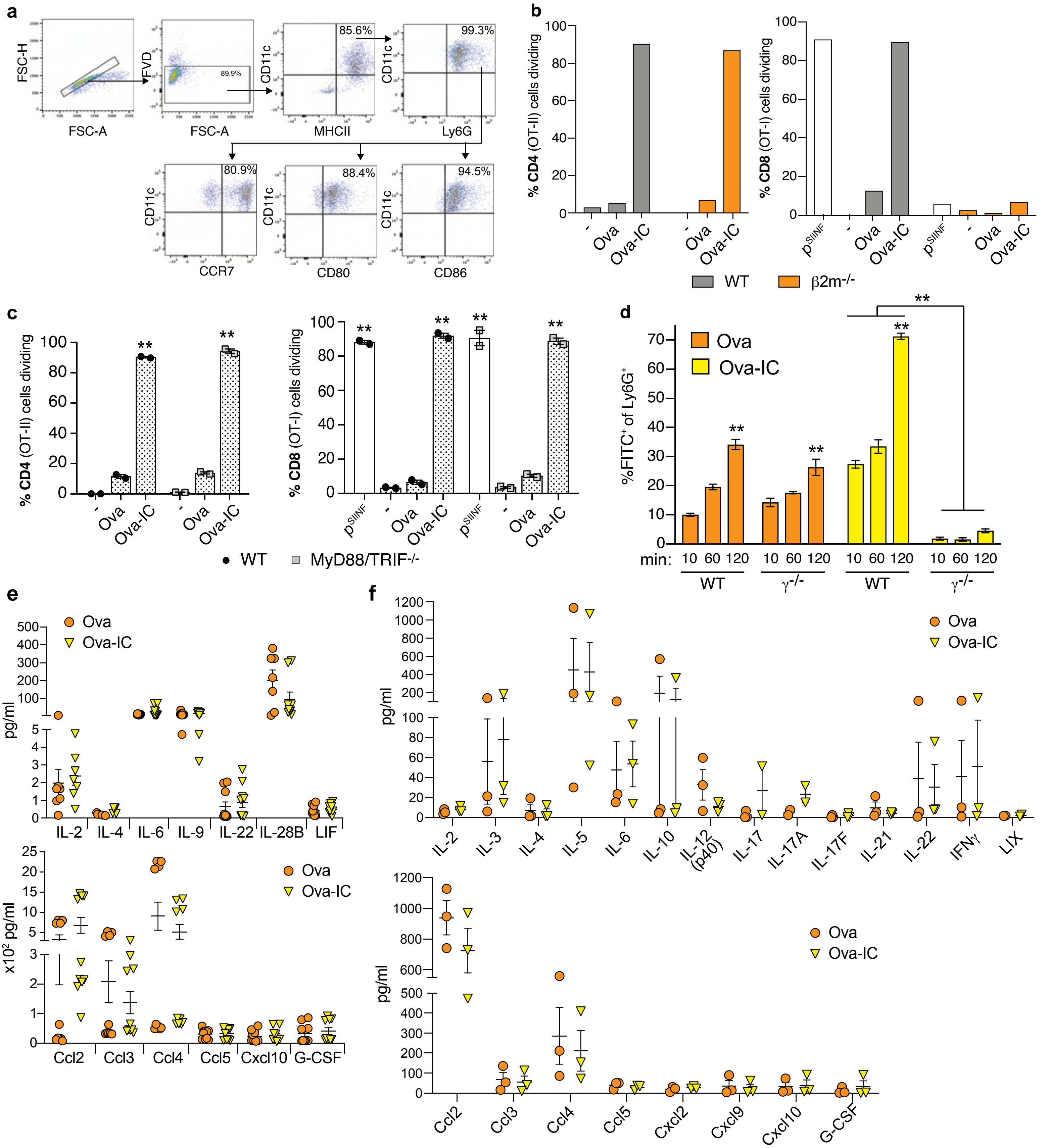

Supplementary Figure 4 (associated with Figure 2). Analysis of T cell proliferation, antigen uptake and APC secreted cytokines. a) Gating strategy for assessing adherent nAPCs from bone marrow neutrophils treated with Ova-IC for $CD11c^{+}MHCII^{+}$ of $Ly6G^{+}$ cells and CD80, CD86 and CCR7. **b)** Percent proliferation of Cell Trace Violet labelled $CD4^{+}$OT-II and $CD8^{+}$OT-I T cells co-cultured with nAPCs derived from WT and β2 microglobulin deficient ($^{-/-}$) mice. As a positive control for OT-I proliferation, GM-CSF generated nAPC were pulsed with the Ova SIINFEKL peptide (p^{SIINF}). **c)** Cell Trace Violet labelled OT-II and OT-I T cells were cultured with Ova or Ova-IC generated nAPCs from WT and $MyD88^{-/-}TRIF^{-/-}$ neutrophils and percent proliferation was calculated. **d)** Percent FITC positive WT and $\gamma^{-/-}$ neutrophils incubated with FITC-Ova or FITC-Ova-IC at indicated times are shown. **e)** Graphs showing the comparable amounts/levels of indicated cytokines in supernatant of Ova-nAPCs and Ova-IC-nAPCs as determined by ELISA. **f)** Cytokine profiles in supernatants of splenic cDCs treated with Ova or Ova-IC. Data is mean ± s.e.m. Significance was determined as follows: c, d) one-way ANOVA and Dunnett's multiple comparison test. *P<0.05 and **P<0.005.

Supplementary Figure 5

a

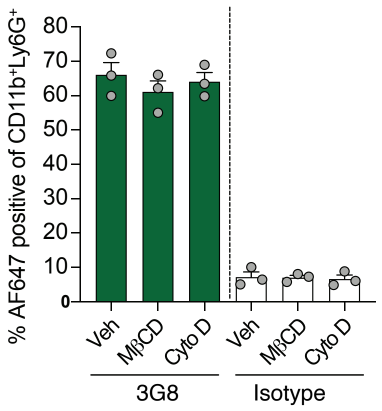

b

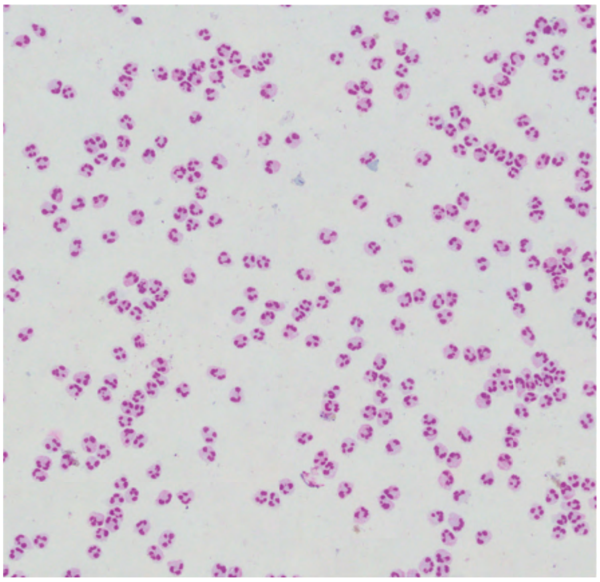

Supplementary Figure 5 (associated with Figure 5). Controls for endocytosis assays and purity of human neutrophils on cyto-spins. a) Isolated 3B/γ$^{-/-}$ neutrophils were treated with vehicle (Veh), Methyl Beta Cyclodextrin (MβCD) or Cytochalasin D (Cyto D) for 30min at 37°C, incubated with FcγRIIIB antibody (3G8) or isotype for 30min at 4°C and then with AF647 labelled anti-mouse secondary to detect 3G8 or isotype (mouse antibodies) on the surface of Ly6G/CD11b. **b)** Representative Wright-Giemsa stained cytospin of neutrophils purified from normal human blood.

Supplementary Figure 6

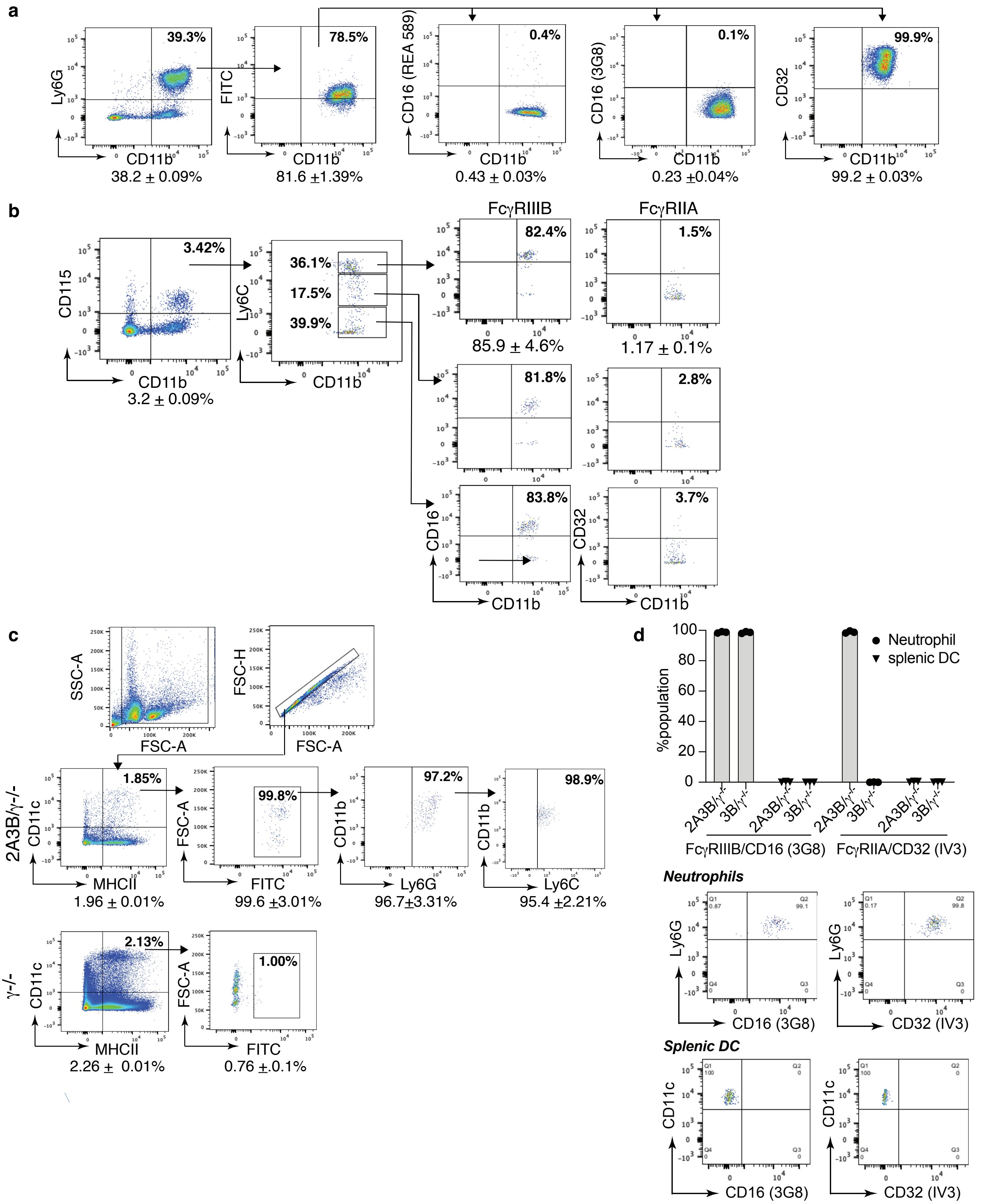

Supplementary Figure 6 (associated with Figure 6). Analysis of human FcγR expression and internalization in FcγR humanized mice. a) Flow plots of surface FcγRIIIB (CD16) and FcγRIIA (CD32) 3.5hrs after i.v. injection of 3G8-fOva conjugate in whole blood. b) Gating strategy for assessing FcγRIIIB (CD16) and FcγRIIA (CD32) expression on CD115+ and Ly6C^{hi} cells in 2A3B/γ$^{-/-}$ mice. c) FACS plots of FITC-positive events in CD11c^{+}MHCII^{+} nAPCs in spleen of 2A3B/γ$^{-/-}$and γ$^{-/-}$ mice 72hrs after 3G8-fOva conjugate injection. d) BMNs and splenic cDCs from 2A3B/γ$^{-/-}$ and 3B/γ$^{-/-}$ mice were analyzed for FcγRIIIB (CD16) and FcγRIIA (CD32) using antibodies 3G8 and IV.3, respectively (upper panel). FACS profile for the same are shown (lower panel).

Supplementary Figure 7

a

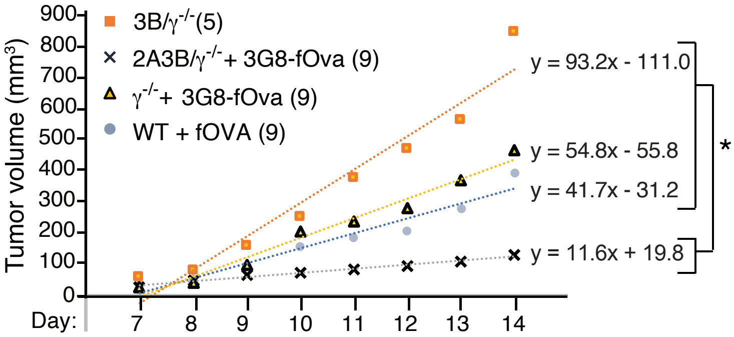

b

CD8

	FVD	CD3/CD8	CD62L^{lo}/ CD44^{hi}	CD62L^{lo}/ CD44^{hi}/Tetr^{+}	CD62L^{lo}/CD44^{hi}/PD1^{+}
fOva	75.74±2.26	13.83±0.45	28.62±6.72	0.61±0.07	0.62±0.15
2A3B/$\gamma^{-/-}$ + 3G8-fOva	81.78±1.08	12.65±0.80	28.14±5.88	3.28±1.11*	1.23±0.31
$\gamma^{-/-}$ + 3G8-fOva	77.08±1.81	12.91±0.54	27.82±5.61	0.42±0.10	0.79±0.20

CD4

	FVD	CD3/CD4	CD4/CD25	CD4/CD69	CD4/Foxp3^{+}	CD4/Ki67^{+}	CD4/Tbet^{+}
fOVA	80.94±4.33	17.28±1.08	0.48±0.11	0.26±0.06	6.31±2.64	13.39±1.87	5.97±0.69
2A3B/$\gamma^{-/-}$ + 3G8-fOVA	85.31±2.34	19.66±0.42	1.30±0.19	0.34±0.1	8.19±2.93	18.56±1.4	5.10±0.49
$\gamma^{-/-}$ + 3G8-fOVA	81.52±3.73	18.20±1.28	1.11±0.17	0.33±0.09	4.56±1.38	16.71±1.57	4.17±1.07

Ratio

	CD8^{+}/Treg^{+}	CD8Tet^{+}/Treg^{+}	CD4 Teff^{+}/Treg^{+}
fOVA	6.69±1.63	0.32±0.09	0.83±0.43
2A3B/$\gamma^{-/-}$ + 3G8-fOva	5.02±1.68	0.92±0.30	0.52±0.18
$\gamma^{-/-}$ + 3G8-fOva	9.01±3.06	0.30±0.11	0.68±0.27

Supplementary Figure 7 (associated with Figure 7). Regression analysis of tumor growth and T cell subset analysis in the B16F10-Ova model. a) Regression analysis was used to compare slope curve co-efficient (tumor growth rate) between the different groups. A Prism regression analysis was used and tested for whether slopes and inter-cepts differ. Overall comparison was done using a one-way ANOVA and Dunnett's multiple comparison. Trend-lines for tumor volume of all groups, created in Excel, are shown. **b)** Table of T cell subset analysis of spleens harvested from mice with transplanted B16F10-Ova cells. Spleens were analyzed for Ova-peptide specific effector CD8 T cells using MHCI-tetramers and markers for CD8 effector (CD62^{lo}CD44^{hi}), CD4 and Treg cells. Results for T cell activation markers (CD25, CD69) proliferation marker (Ki67), transcriptional regulators (T-bet) and PD1 are also shown. *P<0.05 in 2A3B/$\gamma^{-/-}$+3G8-fOva compared to other groups as analyzed by One-way ANOVA and Dunnett's multiple comparison test.

Supplementary Figure 8

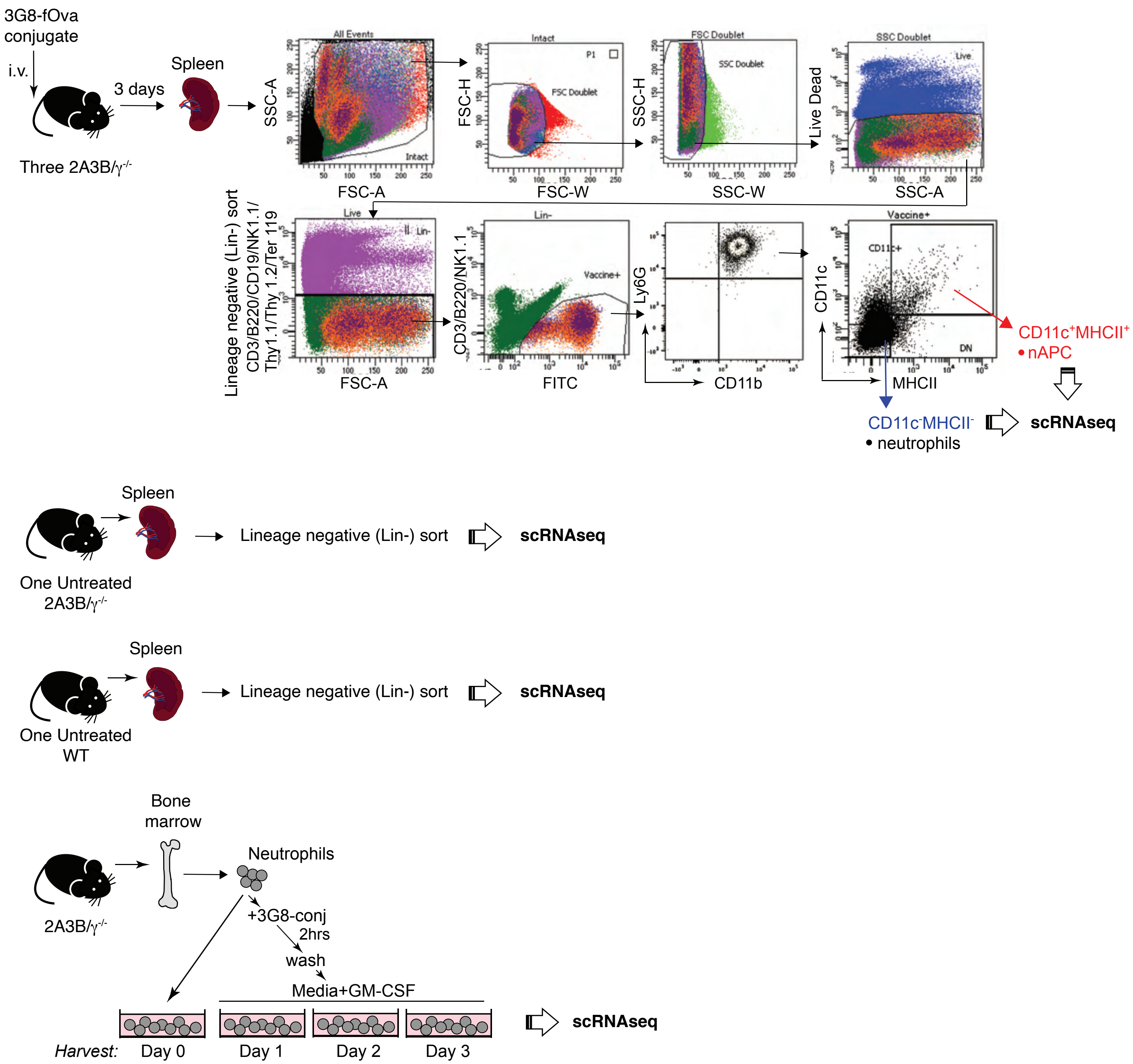

Supplementary Figure 8 (associated with Figure 8). Schematic of study design and analysis of scRNAseq data. Overview of scRNA-seq study design and flow cytometry profiles of sorted populations from spleen.

Supplementary Figure 9

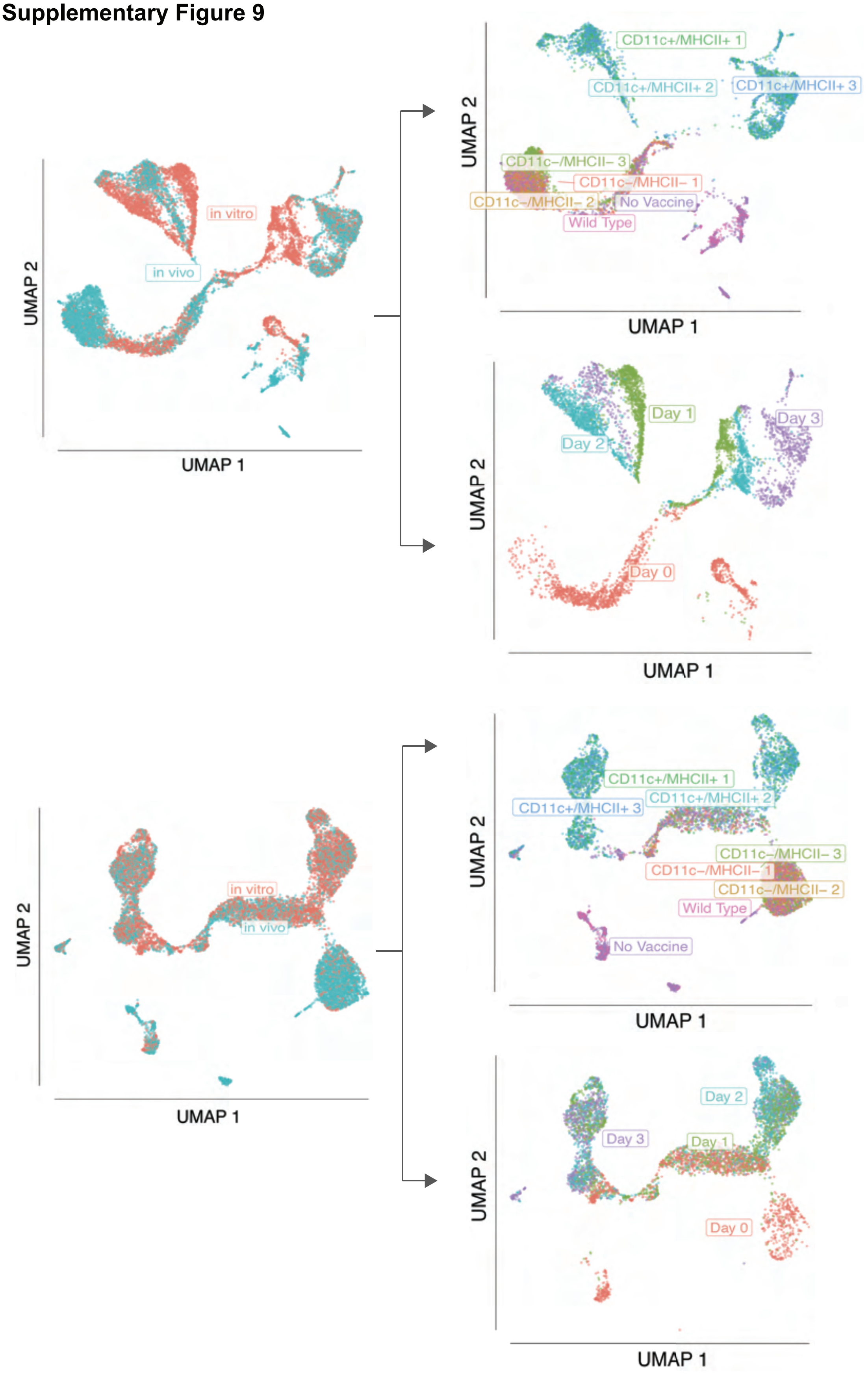

Supplementary Figure 9 (associated with Figure 8). UMAP projection. UMAP projection of data pre-harmonization, colored by in vitro and in vivo (top left) and separated in vivo and in vitro cells colored by hashtag population (top right). UMAP projection of data post-harmonization, colored by in vitro and in vivo (bottom left) and separated in vivo and in vitro cells colored by hashtag population (bottom right).

Supplementary Figure 10

a

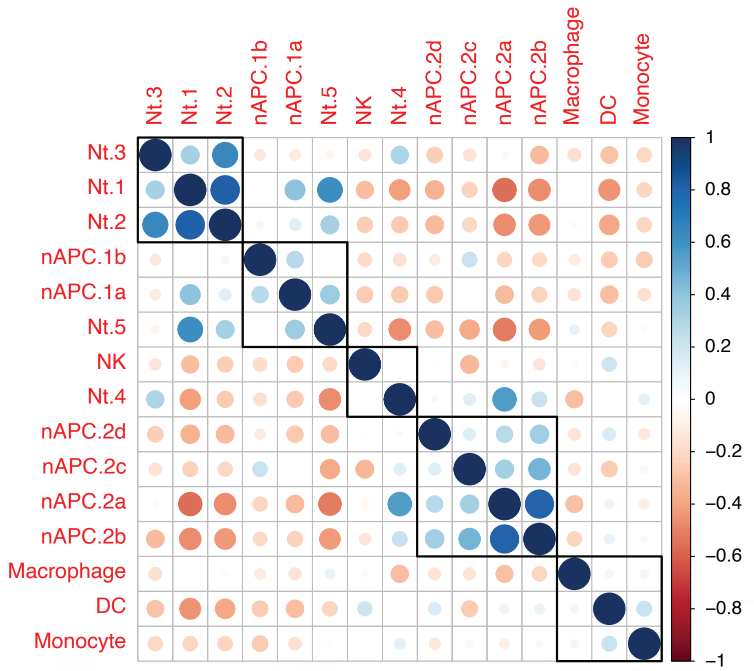

Supplementary Figure 10 (associated with Figure 8). Pseudo-bulk gene correlation and heatmap. a) Pseu-do-bulk (averaged across cell type) gene correlation scores for each identified cell type, grouped into 5 blocks. **b)** Heatmap of top 15 differentially expressed genes across each broad cell type (Nt, nAPC.1 and nAPC.2s collapsed),

b

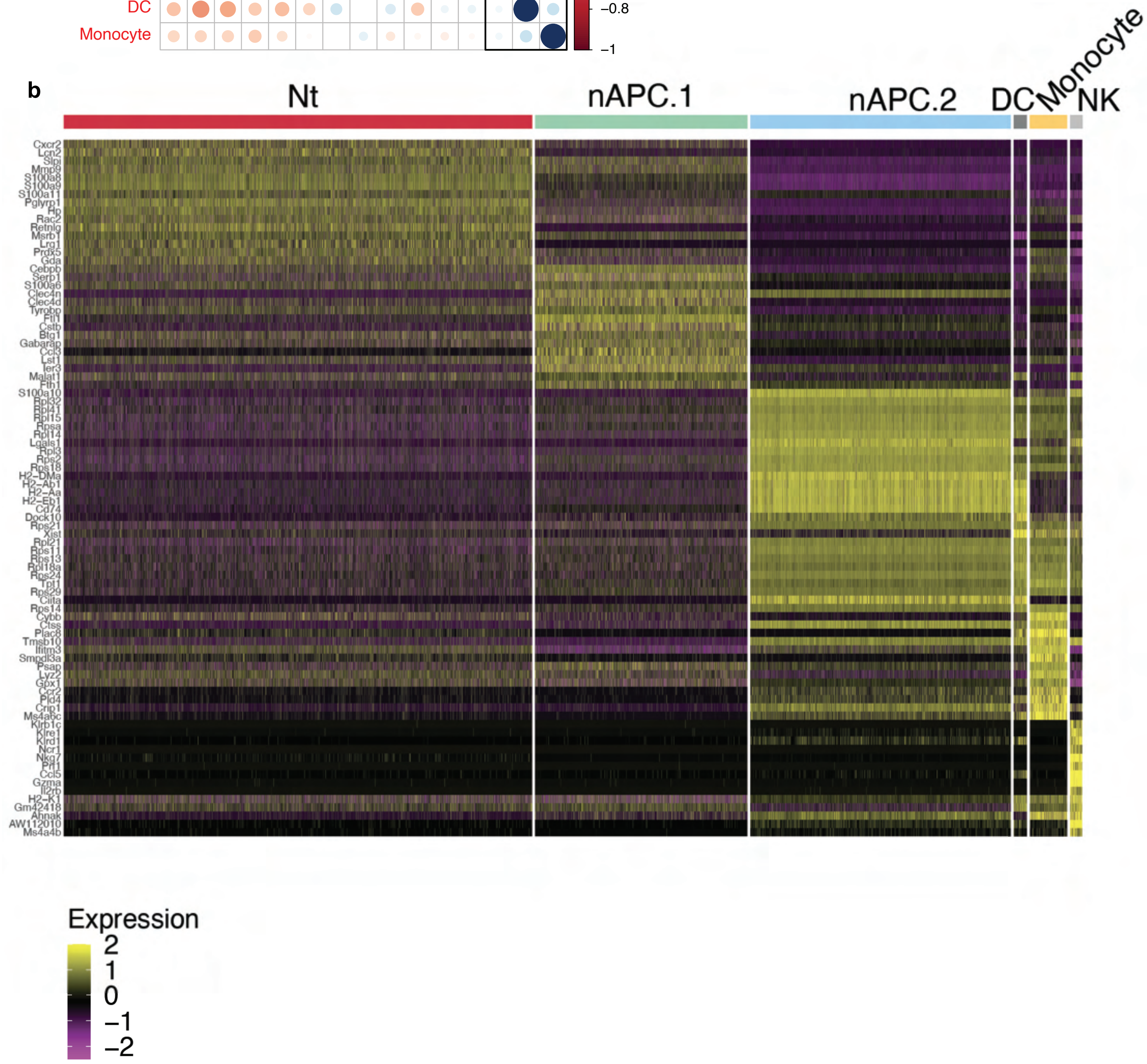

Supplementary Figure 11

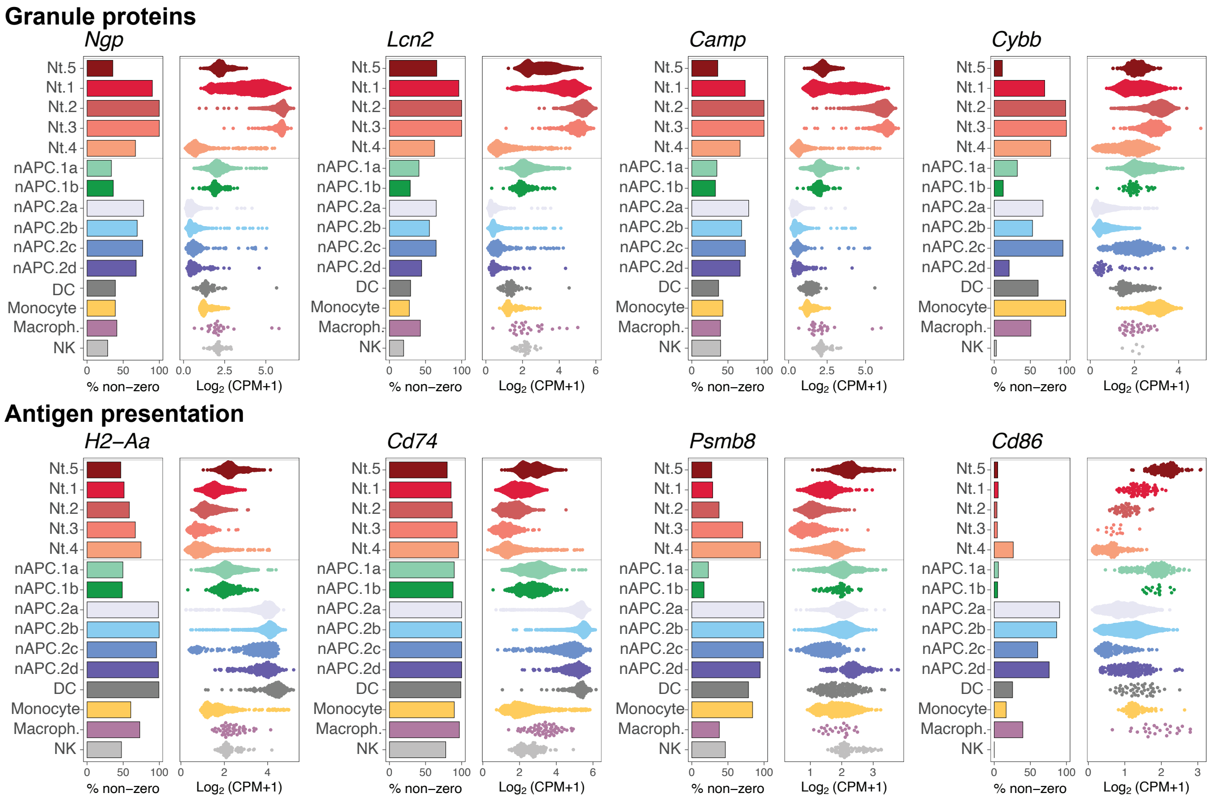

Supplementary Figure 11 (associated with Figure 8). Violin plots of genes. Violin plots of normalized expres-sion of various genes in indicated categories across broad cell types.

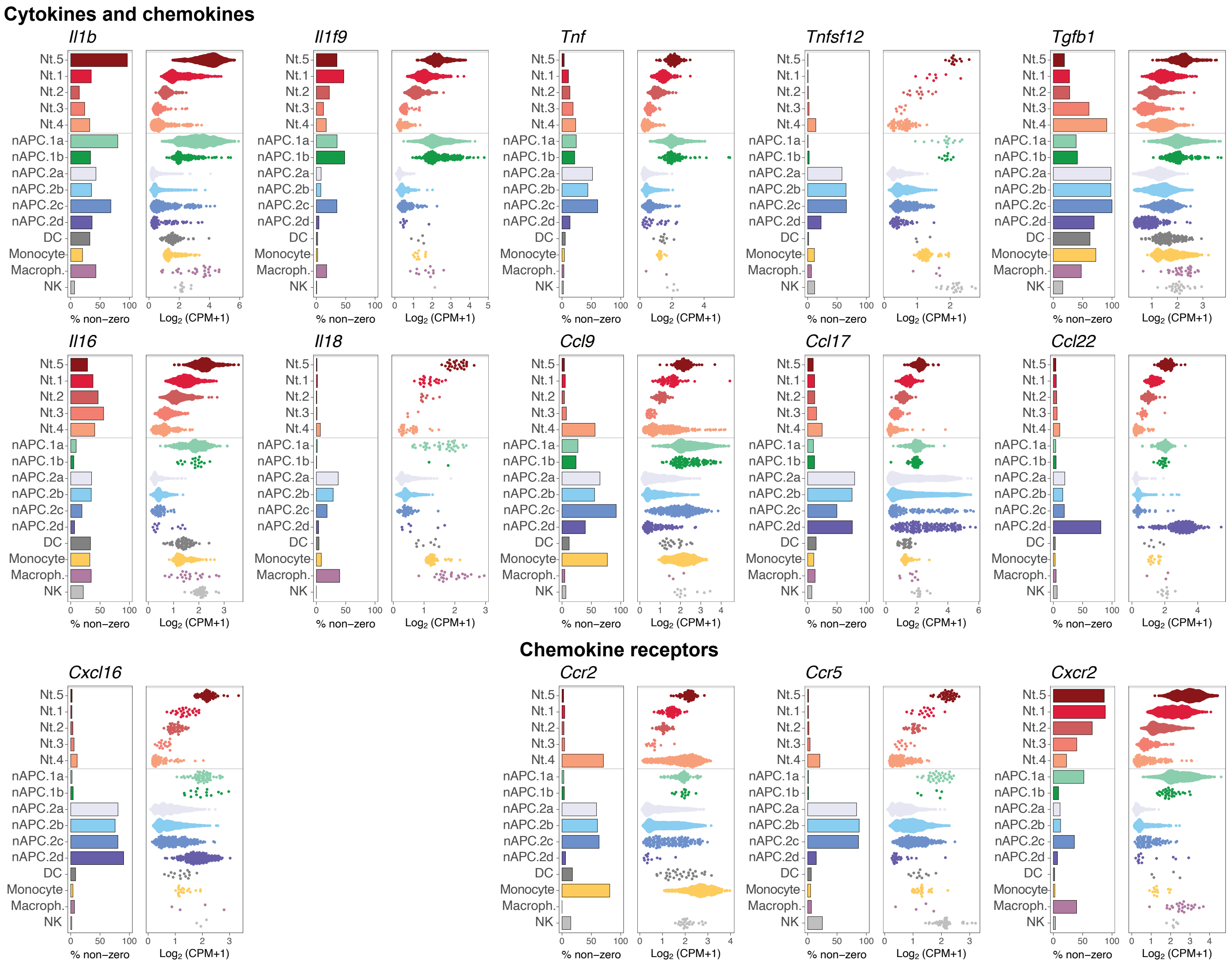

Supplementary Figure 12

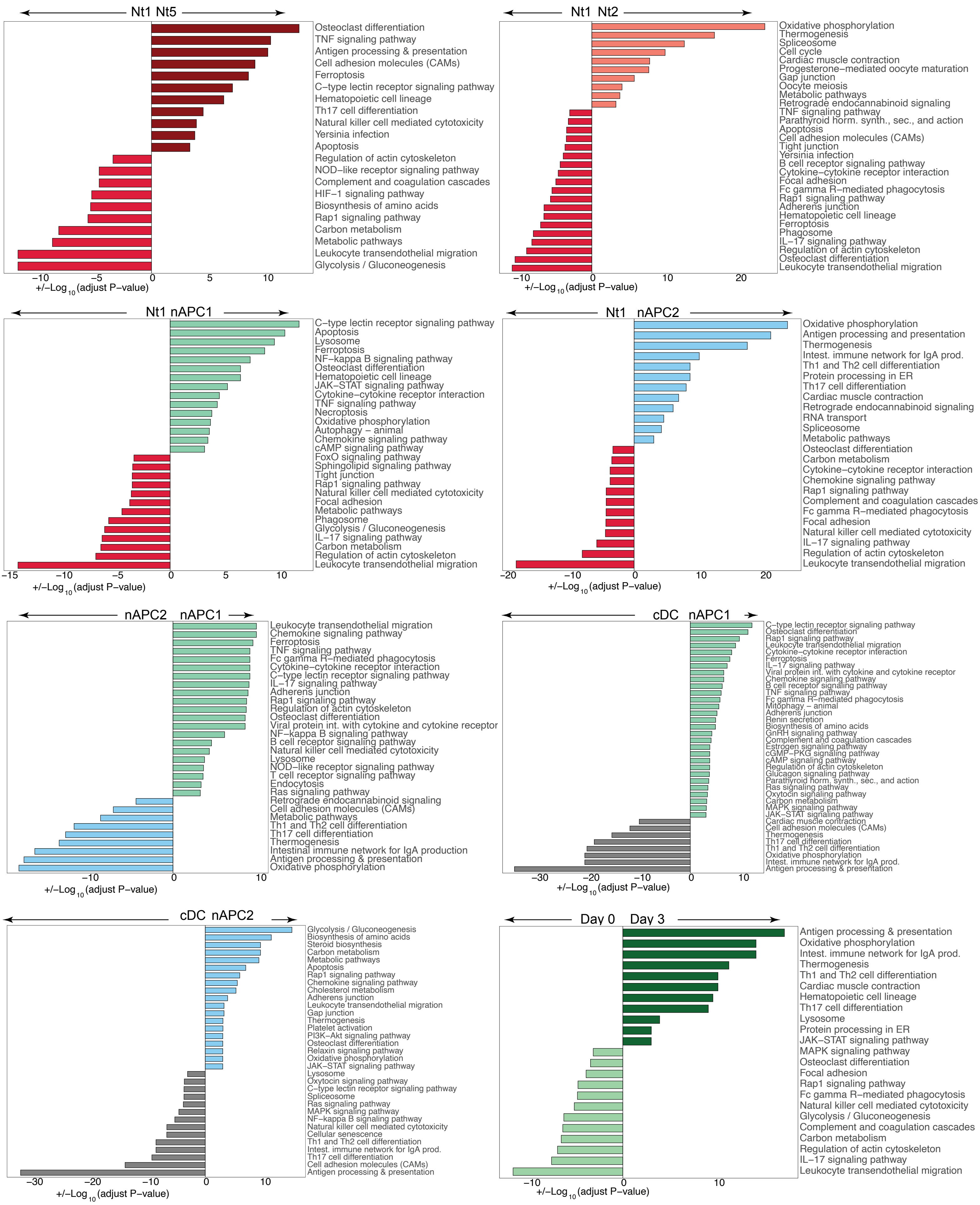

Supplementary Figure 12 (associated with Figure 8). KEGG pathway enrichment. KEGG pathway enrichment p-values for various cell type comparisons.

Supplementary Figure 13

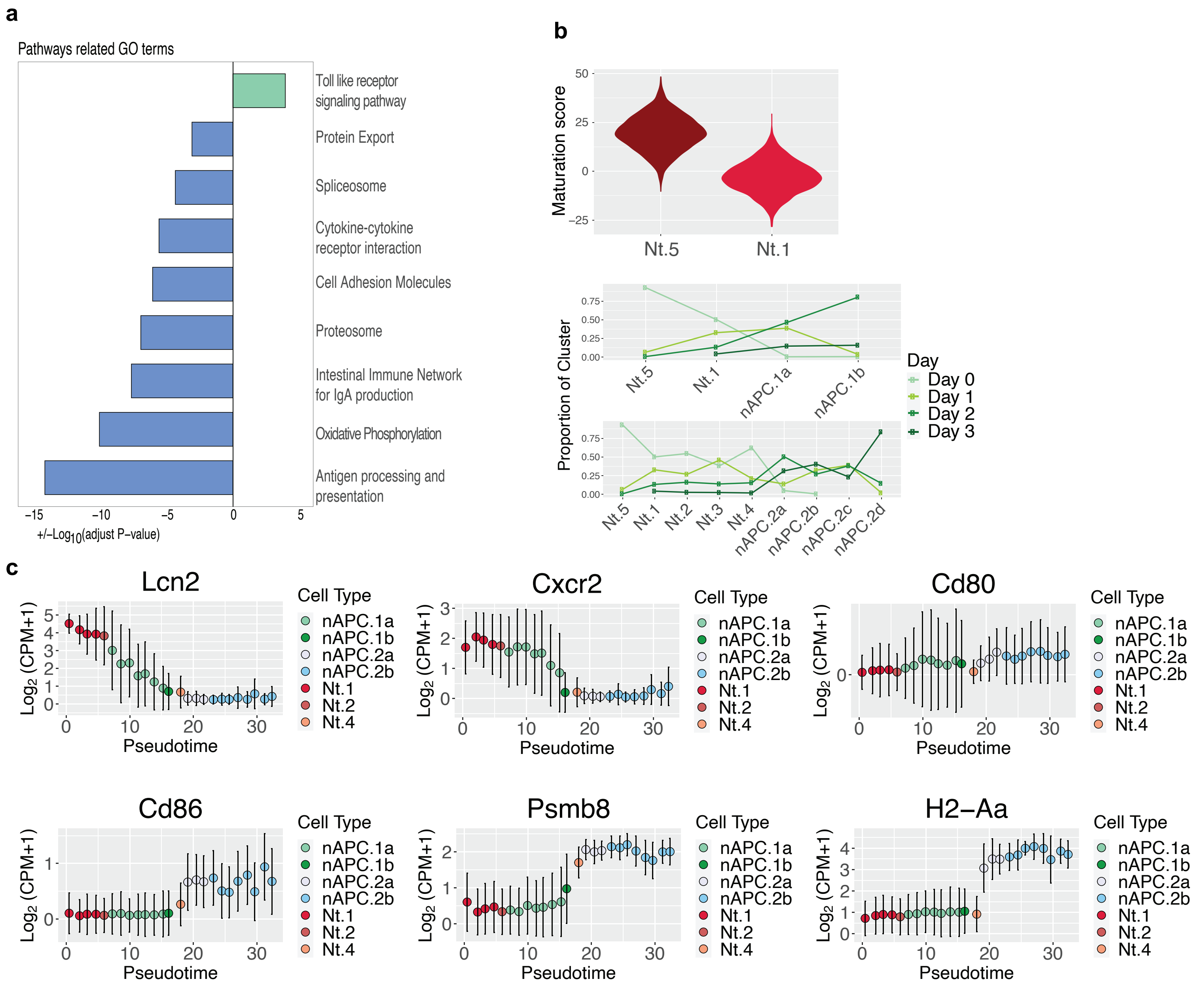

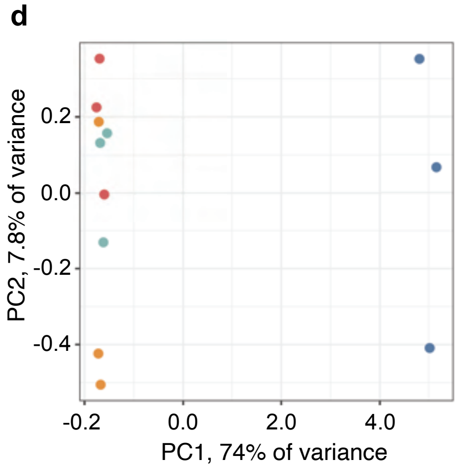

Supplementary Figure 13 (associated with Figure 8). GSEA of variable genes, violin plots and PCA analysis. a) GSEA of all variable genes among nAPC1s (green) and nAPC2s (blue) (Left panel). **b)** Violin plot of maturation score for Nt.1 and Nt.5 subsets (top panel). Proportion of in vitro cells for each cell type split into two potential trajectories (bottom panel). **c)** Mean normalized expres-sion of various markers binned along pseudo-time, d) Plot of PC1 vs. PC2 of PCA analysis on bulk data.

Supplementary Figure 14

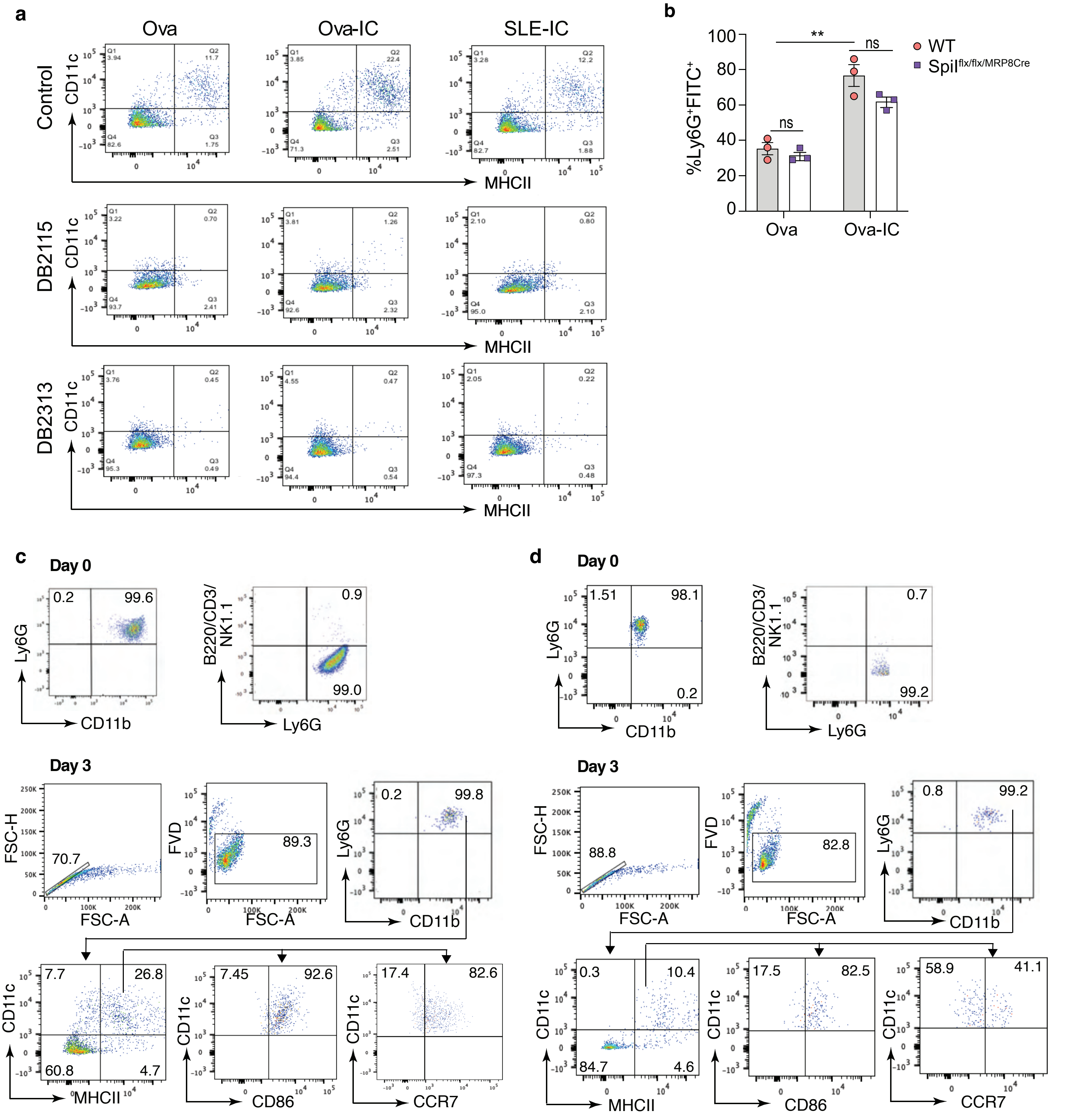

Supplementary Figure 14 (associated with Figure 9). Analysis of wild-type neutrophils treated with PU.1 inhibitor and Spi.1$^{flx/flx/MRP8cre}$ deficient neutrophils. a) Representative profile of neutrophils from 2A3B/γ-/- mice treated with Ova, Ova-IC or SLE-IC and subsequently cultured in vehicle alone (control) or PU.1 inhibitors, DB2115 or DB2313. **b)** Analysis of FITC-Ova- or FITC-Ova-IC uptake by WT and Spi.1$^{flx/flx/MRP8cre}$ deficient neutrophils after 2hrs of incubation. **(c-d)** Flow plots for day 0 neutrophils (top panel) and after day 3 in culture (bottom panel) to examine acquisition of nAPC markers in WT mice (c) and Spi.1$^{flx/flx/MRP8cre}$ (d) mice. Uptake was analyzed using One way ANOVA and Dunnett's multiple comparison test. **p<0.005.

Supplementary Table 1. **Demographic and clinical characteristics of study population**

	**SLE patient (SLE) n=15**	**Normal (N) n=6**
**Female** (%)	13 (86.6%)	4 (66.6%)
**Age**	37.3 ± 4.2	25.6 ± 1.1
**SLEDAI** (mean)	6.26 ± 1.9	
**Nephritis** n (%)	8 (53%)	
**C3** mg/dL	90.6 ± 8.8	
**C4** mg/dL	23.3 ± 3.8	
**dsDNA** IU/mL	84.1 ± 32.2	
**ESR** mm/hr	9.3 ± 4.4	
**CRP** mg/L	0.46 ± 0.15	

Abbreviations: SLE, systemic lupus erythematosus; N, healthy control; SLEDAI, systemic lupus erythematosus disease activity index; dsDNA, double stranded DNA antibodies; ESR, erythrocyte sedimentation rate; CRP, C-reactive protein. Data is average ± SEM.

Supplementary Table 2. Characteristics of patient samples from patients with myeloid neoplasias

Patient	Age/Sex	Diagnosis	Pathogenic variants	Differential cell count
**1**	57M	*Primary myelofibrosis (MPN)*	ATM c.751_752delGT (p.C252*) 60% VAF (787x coverage)<br>JAK2 c.1849G>T (p.V617F) 47.3% VAF (970x coverage)	69% neutrophils<br>6.2% bands<br>9.3% lymphocytes<br>5.4% monocytes<br>0.8% eosinophils<br>3.1% metamyelocytes<br>3.1% myelocytes
**2**	74F	AML<br>arising from myelodysplasia	DNMT3A c.2645G>A (p.R882H) 47.2% VAF (1486x coverage)<br>RUNX1 c.530T>G (p.I177S) 27.5% VAF (641x coverage)<br>SF3B1 c.2098A>G (p.K700E) 38.1% VAF (651x coverage)	27.5% neutrophils<br>18.3% lymphocytes<br>38.9% monocytes<br>14.5% blasts

Supplementary Table 3. Source table for antibodies used for flow cytometry

Source table for murine blood, bone marrow and cultured neutrophils and nAPCs

**Antibody**	**Fluorophore**	**Company**	**Clone**	**Cat#**	**Dilution**
CD3	BV605	BioLegend	17A2	100237	1:100
CD11c	BV605	BioLegend	N418	117334	1:100
F4/80	BV605	BioLegend	BM8	123133	1:100
CD80	BV605	BioLegend	16-10A1	104729	1:100
NK-1.1	BV605	BioLegend	PK136	108739	1:100
Ly-6G	BV605	BioLegend	1A8	127639	1:100
CCR7	Pe/Cy7	BioLegend	4B12	120123	1:100
Ly-6G	PB	BioLegend	1A8	127611	1:100
Ly-6C	PB	BD	AI-21	562727	1:100
CD11b	PB	BioLegend	M1/70	101224	1:100
CD103	PB	BioLegend	2E7	121421	1:100
Ly-6C	PB	BioLegend	HK1.4	128013	1:100
CD86	PB	BioLegend	GL-1	105022	1:100
Ly-6G	Pe/Cy7	BioLegend	1A8	127618	1:100
CD115	Pe/Cy7	BioLegend	AFS98	135524	1:100
CD40	Pe/Cy7	BioLegend	3/23	124622	1:100
CD103	Pe/Cy7	BioLegend	2E7	121425	1:100
CD11b	Pe/Cy7	BioLegend	M1/70	101216	1:100
CD8a	Pe/Cy7	BioLegend	53-6.7	100722	1:100
CD8a	PerCP	BioLegend	53-6.7	100732	1:100
CCR7	PerCP	BioLegend	G043H7	353242	1:100
MHC class II	PerCP	BioLegend	AF6-120.1	116415	1:100
Ly-6G	PerCP	BioLegend	1A8	127616	1:100
Ly-6G	APC	BioLegend	1A8	127614	1:100
CD11c	APC	BioLegend	N418	117310	1:100
Ly-6C	AF647	BioLegend	HK1.4	128009	1:100
CD8a	APC	BioLegend	53-6.7	100711	1:100
CD115	APC	BioLegend	AFS98	135509	1:100
CD11b	Pe	BioLegend	ICRF44	301306	1:100
CD86	Pe	BioLegend	34F	200308	1:100
CCR7	Pe	BioLegend	4B12	120105	1:100
CD11c	Pe	BioLegend	N418	117307	1:100
CD115	Pe	eBioscience	AFS98	12-1152-82	1:100
Ly-6G	FITC	BioLegend	1A8	127606	1:100
CD80	FITC	BioLegend	16-10A1	104705	1:100
CD8a	FITC	BioLegend	5H10-1	100804	1:100
H-2 K^b Ova *Tetramer* (Ova257–264 [*SIINFEKL*]	Pe	NIH			1:600
CD3	BV605	BioLegend	17A2	100237	1:100

Foxp3	PB	BioLegend	MF-14	126419	1:100
CD69	PB	BioLegend	H1.2F3	104527	1:100
CD4	PB	BioLegend	GK1.5	100443	1:100
CD4	PB	BioLegend	RM4-4	116008	1:100
CD69	Pe/Cy7	BD	H1.2F3	552879	1:100
CD25	Pe/Cy7	BioLegend	PC6.1	102016	1:100
CD44	Pe/Cy7	BioLegend	IM7	103030	1:100
CD62l	APC	BioLegend	MEL-14	104411	1:100
Ki67	PerCP	BioLegend	B56	561284	1:100
T-bet	AF647	BioLegend	4B10	644803	1:100
PD-1	APC	BioLegend	RMP1-30	109112	1:100
CTLA-4	APC	BioLegend	UC10-4B9	106310	1:100
CD3	APC	BioLegend	145-2C11	100311	1:100
PD-1	Pe	BioLegend	RMP1-14	114117	1:100
MHC class II	Pe	eBioscience	NIMR-4	12-5322-81	1:100
CD25	PE-CF594	BD	PC61	562695	1:100

Supplementary Table 4. Source table for antibodies used for flow cytometry

Source Table for human blood and cultured blood neutrophils and nAPC

Antibody	Fluorophore	Company	Clone	Cat#	Dilution
CD80	APC-H7	BD	L307.4	561134	1:100
XCR1	Pe	BioLegend	S15046E	762603	1:100
CD86	BV421	BioLegend	IT2.2	305426	1:100
CD141	Pe/Cy7	BioLegend	M80	344109	1:100
CD11c	BV421	BD	3.9	565807	1:100
CD14	APC	BioLegend	M5E2	301808	1:100
CD15	BV605	BioLegend	W6D3	323031	1:100
CD10	Pe	Ancell Corporation	SN5c/L4-1A1	157-050	1:100
SiglecH	Pe	eBioscience	eBio440c	12-0333-80	1:100
CD11c	APC/Cy7	BioLegend	Bu15	337218	1:100
CD11b	Pe	BioLegend	ICRF44	301306	1:100
HLA-DR	Bv605	BioLegend	L243	307639	1:100
Clec9a	AF488	R&D Systems	683409	FAB6049G	1:100
CD10	BV421	BD	H10a	562902	1:200
CD11c	AF647	BioLegend	3.9	301619	1:100
CD197	BV421	BD	150503	562555	1:100
CD80	Pe/Cy7	BioLegend	2D10	305218	1:100
CD86	PerCP	BioLegend	IT2.2	305419	1:100
CD11b	Pe/Cy7	BioLegend	M1/70	101216	1:100
CD15	PerCP	BioLegend	W6DE	323020	1:100
Clec9A	Pe	BioLegend	8F9	353803	1:100
CD16	Pe	BD	3G8	555407	1:100
CD32	Pe	BD	FLI8.26	550586	1:100
CD16	APC Vio770	MACS-Myltenyi Biotech	REA589	130-109-147	1:100

Supplementary Table 5. Source table for antibodies used for flow cytometry

Source table for normal and SLE patient blood samples

Antibody	Fluorophore	Company	Clone	Cat#	Dilution
CD11c	PE	BioLegend	301606	3.9	1:20
CD10	APC/Cy7	BioLegend	312212	HI10A	1:20
CD15	FITC	BioLegend	301903	HI98	1:100
HLA-DR	PE/Cy7	BioLegend	307616	L243	1:100
CD3	PerCP/Cy5.5	BioLegend	300328	HIT3a	1:100
CD19	PerCP/Cy5.5	BioLegend	302230	HIB19	1:100
CD56	PerCP/Cy5.5	BioLegend	318322	HCD56	1:100
CD14	Pacific Blue	BioLegend	301828	M5E2	1:100
CD197 (CCR7)	Pacific Blue	BioLegend	353210	G043H7	1:100
CD80	APC	BioLegend	305220	2D10	1:100
CD141	BV605	BD OptiBuild	740421	1A4	1:100
CD370 (CleC9)	APC	BioLegend	353806	8F9	1:100

Supplementary Table 6. Source table for antibodies used for flow cytometry

Isotype controls and Fc block reagents

Human TruStain FcX	Biolegend	422302
Mouse BD Fc Block	BD	553141
Mouse IgG1k	BD	557273
Mouse IgG1k-FITC	Invitrogen	11-4714-81
