# Supplementary material for: FcγR engagement reprograms neutrophils into antigen cross-presenting cells that elicit acquired anti-tumor immunity
Source: Nat Commun. 2021 Aug 9;12:4791. doi: 10.1038/s41467-021-24591-x (PMC8352912; doi:10.1038/s41467-021-24591-x)
Supplement: Supplementary file 3 — Reporting Summary [file 41467_2021_24591_MOESM3_ESM.pdf]

nature research

Corresponding author(s): Tanya N. Mayadas

Last updated by author(s): Jun 6, 2021

Reporting Summary

Nature Research wishes to improve the reproducibility of the work that we publish. This form provides structure for consistency and transparency in reporting. For further information on Nature Research policies, see our Editorial Policies and the Editorial Policy Checklist.

Statistics

For all statistical analyses, confirm that the following items are present in the figure legend, table legend, main text, or Methods section.

n/a	Confirmed	
☐	☒	The exact sample size (*n*) for each experimental group/condition, given as a discrete number and unit of measurement
☐	☒	A statement on whether measurements were taken from distinct samples or whether the same sample was measured repeatedly
☐	☒	The statistical test(s) used AND whether they are one- or two-sided<br>*Only common tests should be described solely by name; describe more complex techniques in the Methods section.*
☐	☒	A description of all covariates tested
☐	☒	A description of any assumptions or corrections, such as tests of normality and adjustment for multiple comparisons
☐	☒	A full description of the statistical parameters including central tendency (e.g. means) or other basic estimates (e.g. regression coefficient) AND variation (e.g. standard deviation) or associated estimates of uncertainty (e.g. confidence intervals)
☐	☒	For null hypothesis testing, the test statistic (e.g. *F*, *t*, *r*) with confidence intervals, effect sizes, degrees of freedom and *P* value noted<br>*Give P values as exact values whenever suitable.*
☒	☐	For Bayesian analysis, information on the choice of priors and Markov chain Monte Carlo settings
☒	☐	For hierarchical and complex designs, identification of the appropriate level for tests and full reporting of outcomes
☐	☒	Estimates of effect sizes (e.g. Cohen's *d*, Pearson's *r*), indicating how they were calculated

Our web collection on statistics for biologists contains articles on many of the points above.

Software and code

Policy information about availability of computer code

Data collection	Flow collection was performed using FACSDIVA (BD Pahrmingen). Sequencing data was collected on a NextSeq 500 (Illumina) and ELISPOT data was acquired using Immunospot software (Cellular Technology limited). For live cell imaging Nikon NIS Elementens AR 4.30 and MetaMorph 7.8 (Molecular Devices) was used. For intravital microscopy Prairie View Software (Bruker Technology) software was used for data collection.
Data analysis	ELISPOT results was analyzed using CTL Immunospot analyzer. All flow data was analyzed using Flowjo version 10.5. For microscope image analysis Fiji or Imaris (bitplane) was used. Cellranger v3.0.2 was used to analyze raw files for sequencing studies. STATA 13, JMP10 was widely used for statistics. For smaller scale experiments Graphpad prism 8 was used.

For manuscripts utilizing custom algorithms or software that are central to the research but not yet described in published literature, software must be made available to editors and reviewers. We strongly encourage code deposition in a community repository (e.g. GitHub). See the Nature Research guidelines for submitting code & software for further information.

Data

Policy information about availability of data

All manuscripts must include a data availability statement. This statement should provide the following information, where applicable:

- Accession codes, unique identifiers, or web links for publicly available datasets
- A list of figures that have associated raw data
- A description of any restrictions on data availability

The data and materials that support the findings of this study are available from the corresponding author upon reasonable request. All data presented on graphs are presented as individual data points. Source data underlying the summary figure 4d,4e,6a,7a and 7b has been provided as a source data file.

Field-specific reporting

Please select the one below that is the best fit for your research. If you are not sure, read the appropriate sections before making your selection.

- [x] Life sciences
- [ ] Behavioural & social sciences
- [ ] Ecological, evolutionary & environmental sciences

For a reference copy of the document with all sections, see nature.com/documents/nr-reporting-summary-flat.pdf

Life sciences study design

All studies must disclose on these points even when the disclosure is negative.

Sample size	Sample size was chosen based on experience to ensure the possibility of statistical analysis and to minimize the use of animals according to the BWH/IACUC standing committee .
Data exclusions	No data was excluded
Replication	All results in the paper are drawn from the analysis of multiple animals (between 3 and 10) per experiment. Small scale experiments were performed in triplicates with good correspondence between replicates.
Randomization	Age and sex-matched animals were used for each experiment. Animals were also co-housed when possible.
Blinding	The inflammatory score for Delayed type hypersensitivity response was analyzed by an observer blinded to the identity of the samples. All tumor experiments were blinded during data collection

Reporting for specific materials, systems and methods

We require information from authors about some types of materials, experimental systems and methods used in many studies. Here, indicate whether each material, system or method listed is relevant to your study. If you are not sure if a list item applies to your research, read the appropriate section before selecting a response.

Materials & experimental systems

n/a	Involved in the study	
[ ]	[x]	Antibodies
[ ]	[x]	Eukaryotic cell lines
[x]	[ ]	Palaeontology and archaeology
[ ]	[x]	Animals and other organisms
[ ]	[x]	Human research participants
[x]	[ ]	Clinical data
[ ]	[ ]	Dual use research of concern

Methods

n/a	Involved in the study	
[x]	[ ]	ChIP-seq
[ ]	[x]	Flow cytometry
[x]	[ ]	MRI-based neuroimaging

Antibodies

Antibodies used	Detailed description of antibody including the Catalog number, clone and manufacturer are provided in a table format in the manuscript. Additionally, Anti-BSA rabbit antibody (#B7276) and anti-Ova rabbit antibody (#C6534) were form Sigma Aldrich. Anti-NIP human IgG1 (chimeric antibody with lambda light mouse chains and heavy human chains) was a gift from Richard Blumberg (Brigham and Women's Hosp, Boston).H-2 Kb Ova Tetramer(Ova257–264) was from the NIH Tetramer Core Facility. Anti-FcgRIIIB (3G8) (Biolegend) was conjugated to FITC-Ova (#O23020, Thermofisher) as a custom order (Biolegend) and yielded species of various molecular weights indicating the presence of complexes of different 3G8 to fOva ratios.<br>Immune complexes were prepared from anti-Ova, anti-NIP-Ova or anti-BSA antibodies by mixing Ova, NIP-Ova or BSA at a 1:1 ratio.
Validation	Titrations of FACS antibodies was performed based on manufacturer's recommendations and confirmed in the data sheets provided by the manufacturer.

Eukaryotic cell lines

Policy information about cell lines

Cell line source(s)	B16F10-OVA was created in the lab of Dr Arlene Sharpe, Harvard Medical School and was a kind gift.
Authentication	B16F10-OVA was validated in the lab of Dr. Arlene Sharpe.
Mycoplasma contamination	Cell line was confirmed to be mycoplasma negative.

Commonly misidentified lines (See ICLAC register)	n/a

Animals and other organisms

Policy information about studies involving animals; ARRIVE guidelines recommended for reporting animal research

Laboratory animals	Details on the mice used in this study have been included in the Methods section of this manuscript.<br>Wild-type mice, FcRg-/-, g -/- mice expressing human FcgRs (FcgRIIA(2A)/g-/-, FcgRIIIB(3B)/g-/- and FcgRIIA+IIIB(2A3B)/g-/-), B2m-/- (The Jackson Laboratory), MyD88/TRIF-/-, B6.SJL-Ptprca Pepcb/BoyJ (The Jackson Laboratory), CD11c-YFP (B6.Cg-Tg(Itgax-Venus) 1Mnz/J, The Jackson Laboratotry), Granulocyte-specific PU.1 conditional knock-out mice, MRP8-Cre-IRES/GFP-Spi1f/f 63, OT-I, expressing the transgenic T cell receptor recognizing Ovalbumin residues 257-264 (SIINFEKL) in the context of H2Kb (The Jackson Laboratory), OT-I/ beta actin-GFP, obtained by crossing OT-I mice with beta actin-GFP mice, OT II, transgenic mice expressing alpha and beta-chain T cell receptor recognizing Ovalbumin 323-339 peptide in the context of H2Ka and B-actin RFP (The Jackson Laboratory). Both female and male mice were included in the study and was used at age 9-12 weeks.
Wild animals	Study did not involve wild animals.
Field-collected samples	Study did not involve samples collected from the field.
Ethics oversight	The Brigham and Women's hospital animal care and use committee.

Note that full information on the approval of the study protocol must also be provided in the manuscript.

Human research participants

Policy information about studies involving human research participants

Population characteristics	Blood samples were drawn from consented normal human volunteers or patients with myeloid neoplasia or lupus nephritis of all ages and both genders.
Recruitment	Blood samples were obtained from healthy controls with no history of auto-immune diseases and patients with SLE who fulfilled the 1997 ACR classification criteria.
Ethics oversight	Brigham and Women's hospital Institutional Review Board (P001694/PHS) and Dana Farber Cancer Institute Institutional review board (01-206) and IRB of the Instituto Nacional de Ciencias Médicas y Nutrición Salvador Zubirán (IRE-2297)

Note that full information on the approval of the study protocol must also be provided in the manuscript.

Dual use research of concern

Policy information about dual use research of concern

Hazards

Could the accidental, deliberate or reckless misuse of agents or technologies generated in the work, or the application of information presented in the manuscript, pose a threat to:

No	Yes	
☒	☐	Public health
☒	☐	National security
☒	☐	Crops and/or livestock
☒	☐	Ecosystems
☒	☐	Any other significant area

Experiments of concern

Does the work involve any of these experiments of concern:

No	Yes	
☒	☐	Demonstrate how to render a vaccine ineffective
☒	☐	Confer resistance to therapeutically useful antibiotics or antiviral agents
☒	☐	Enhance the virulence of a pathogen or render a nonpathogen virulent
☒	☐	Increase transmissibility of a pathogen
☒	☐	Alter the host range of a pathogen
☒	☐	Enable evasion of diagnostic/detection modalities
☒	☐	Enable the weaponization of a biological agent or toxin
☒	☐	Any other potentially harmful combination of experiments and agents

Flow Cytometry

Plots

Confirm that:

☒ The axis labels state the marker and fluorochrome used (e.g. CD4-FITC).

☒ The axis scales are clearly visible. Include numbers along axes only for bottom left plot of group (a 'group' is an analysis of identical markers).

☒ All plots are contour plots with outliers or pseudocolor plots.

☒ A numerical value for number of cells or percentage (with statistics) is provided.

Methodology

Sample preparation	Leukocytes isolated from mice and human peripheral blood.
Instrument	FACSCanto II; LSRFortessa-12-color; FACSSymphony.
Software	Flow cytometry data was collected with BD FACS Diva software and analyzed with FlowJo version 10.5 software.
Cell population abundance	All relevant information is provided in the manuscript.
Gating strategy	A detailed description of gating strategy along with FACS plots are provided for every experiment in the supplementary information. Doublets were removed using Forward and side scatter (height and area). Viable cells were gated for specific markers to identify different cells types. Gates are drawn based on single-stain and full-minus-one (FMO) controls.

☒ Tick this box to confirm that a figure exemplifying the gating strategy is provided in the Supplementary Information.
