# Supplementary material for: FcγR engagement reprograms neutrophils into antigen cross-presenting cells that elicit acquired anti-tumor immunity
Source: Nat Commun. 2021 Aug 9;12:4791. doi: 10.1038/s41467-021-24591-x (PMC8352912; doi:10.1038/s41467-021-24591-x)
Supplement: Supplementary file 4 — Description of Additional Supplementary Files [file 41467_2021_24591_MOESM4_ESM.pdf]

Description of Additional Supplementary Files

Supplementary Data 1. Single cell RNAseq: Differentially expressed genes (DEGs) for each identified cell type cluster compared to all other clusters.

Supplementary Data 2. Pseudo-bulk RNAseq: Differentially expressed genes (DEGs) for nAPC1 vs. nAPC2.

Supplementary Data 3. List of genes used for trajectory score and maturation score.

Supplementary Data 4. Bulk RNAseq: Differentially expressed genes (DEGs) for each condition pairwise.

Supplementary Movie 1. Day 0 and Day 2 of peripheral blood neutrophils stimulated with SLE-IC imaged in Cy5 fluorescence filter set. SYTO™ stained, peripheral blood neutrophils from CD11c-YFP mice stimulated with SLE-IC were washed and cultured for 2 days. Z-stack images were acquired at day 0 and 2 by confocal microscopy using Cy5 fluorescence filter sets. Open arrowheads, neutrophils.

Supplementary Movie 2. Day 0 and Day 2 of peripheral blood neutrophils stimulated with SLE-IC imaged in Cy5 fluorescence filter set. SYTO™ stained, peripheral blood neutrophils from CD11c-YFP mice stimulated with SLE-IC were washed and cultured for 2 days. Z-stack images were acquired at day 0 and 2 by confocal microscopy using YFP fluorescence filter sets. Closed arrows, YFP+ cells that are mononuclear in Supplementary Movie/Audio/Data 1 as also indicated in Figure 1f; Open arrows, YFP low cells that are mononuclear in Supplementary Movie/Audio/Data 1 that are not visible in Figure 1f.

Supplementary Movie 3. Live cell imaging of peripheral blood neutrophils stimulated with SLE-IC. SYTO stained, highly purified peripheral blood neutrophils from CD11c-YFP mice were stimulated with for 1.5hrs in a microfluidic device with traps, washed with media and imaged by confocal microscopy. Imaging commenced from the start of SLE-IC stimulation. Two z-stacks in Cy5 (with exception of first timepoint, which has 3 z-stacks) and one each in brightfield (BF) and YFP are shown. Note that the nuclear changes in Cy5 are also observed in brightfield (BF).

Supplementary Movie 4. Intravital microscopy of interactions between adoptively transferred nAPCs and CD8 T cells in the popliteal lymph node. OT-I-GFP CD8+ T cells (green) cluster around and make prolonged contacts with RFP-nAPCs (red), which extend long processes.

Supplementary Movie 5. Intravital microscopy of adoptively transferred nAPCs and CD8 T cells in the popliteal lymph node. In areas with no RFP-nAPCs, few OT-I-GFP CD8+ T cells (Green) are observed and they do not cluster.
